# Heparanase (*HPSE*) gene polymorphism (rs12503843) contributes as a risk factor for hepatocellular carcinoma (HCC): a pilot study among Egyptian patients

**DOI:** 10.1186/s43141-020-00106-x

**Published:** 2021-01-07

**Authors:** Faten Saad, Mahmoud Gadallah, Ahmed Daif, Nahed Bedair, Moustafa A. Sakr

**Affiliations:** Molecular Diagnostics and Therapeutics Department, Genetic Engineering and Biotechnology Research Institute (GEBRI), University of Sadat City, Sadat City, Egypt

**Keywords:** Single nucleotide polymorphisms, *HPSE* gene, Hepatocellular carcinoma, ARMS PCR, PCR-RFLP

## Abstract

**Background:**

Heparanase activity was found to be included in human cancer development and growth. Heparanase (*HPSE*) gene single nucleotide polymorphisms (SNPs) have been found to be correlated with different human cancers. In the current study, we investigated whether *HPSE* SNPs were a hepatocellular carcinoma (HCC) risk factor by carrying out a comprehensive case-control pilot study. *HPSE* rs12331678 and rs12503843 were genotyped in 70 HCC-diagnosed patients and 30 healthy controls by modified amplification refractory mutation system (ARMS PCR) and polymerase chain reaction-restriction fragment length polymorphism (PCR-RFLP) analysis.

**Results:**

*HPSE* rs12331678 distributions showed that there were no statistically significant differences between both cohorts either in genotypic or allelic distribution but there was a significant correlation between the rs12503843 (T allele) and the HCC risk in the whole samples (*P* = 0.042). No significant association was observed between the *HPSE* rs12331678 and rs12503843 gene polymorphisms and all clinicopathologic markers or with SNP stratification based on HCV carrier in HCC groups.

**Conclusion:**

Our findings suggest for the first time the *HPSE* gene SNP characterization in HCC Egyptian patients, and our findings reveal there were associations between the *HPSE* rs12503843 (T allele) and the susceptibility to HCC.

## Background

Human hepatocellular carcinoma (HCC) is one of the leading cancer-related causes of death; it is a common malignancy in developing countries with rising incidence as it has a high prevalence in Southeast Asia, China, and sub-Saharan Africa with low incidence in the USA and Europe [1]. The process of HCC carcinogenesis is complex and multistep. Several risk factors are believed to have hepatocarcinogenesis contribution, such as chronic hepatitis C and B virus (HCV, HBV) infection, cirrhosis, carcinogen exposure, and many single nucleotide polymorphisms (SNPs) [2–5]. The HCC progression and metastasis mechanisms are still not fully clarified. Moreover, the prognosis of HCC is still poor due to tumor cell frequent intrahepatic spread, invasiveness, and metastasis [6].

Heparanase, a mammalian endo-β-D-glycosidase, particularly cleaves the heparin glycosaminoglycans sulfate side chains, the most abundant basement membrane and extracellular matrix macromolecules [7]. Heparanase activity can influence several biological and pathological processes, including tissue repair, inflammation, tumor angiogenesis, invasion, and metastasis [8, 9]. Different studies have studied the clinical significance of heparanase in patients with HCC using immunohistochemistry, RT-PCR and qPCR, in situ hybridization, tissue microarrays (TMAs), and western blotting with upregulation in HCC [10–14]. Heparanase overexpression was recognized in a growing number of human primary tumors, associating with increased distant or recurrence metastasis and increased micro-vessel density [15, 16].

Downregulating heparanase expression either by using RNA interference or antisense oligodeoxynucleotides significantly reduces the invasiveness, angiogenesis, and metastasis of human HCC SMMC7721 cell line [17]. Two anti-heparanase antibodies (multiple antigenic peptides MAP1-2) can effectively prevent the heparanase activity of hepatic cancer cells (HCCLM6), thereby affecting their invasive capability and indicating their pivotal role in HCC tumor growth and metastasis [18].

Single nucleotide polymorphisms (SNPs) are the most abundant DNA variation sequence. It arises when one nucleotide in the shared nucleotide sequences of a specific gene varies between species members or in combined chromosomes. When the SNP is found within the regulatory sequences of the gene, the expression of this gene can be affected which in turn be correlated with the occurrence and progression of specific diseases [19–22]. Several previous reports suggested that SNPs of *HPSE* are accompanying with different types of cancers, such as hematological malignancies, gastric cancer, and ovarian carcinoma [23–28].

Therefore, to clarify the multifactorial and biological behavior of hepatocarcinogenesis and expand the scientific background for protective mediations, SNP identification or combined interaction of different SNPs in specific genes might be helpful in HCC. The role of heparanase and the prognosis in human malignancy has been well studied, but the role of *HPSE* polymorphisms in HCC is still controversial. We hypothesized that heparanase polymorphisms could play a vital role in the development of HCC. Hence, in this study, we conducted a case-control study of heparanase SNPs located in this gene to analyze the contribution of the polymorphisms of heparanase with the susceptibility or pathological development of HCC in Egyptian patients.

## Methods

### Study population

Seventy HCC cases and thirty control subjects were recruited from National Liver Institute, Menoufia University, Egypt (controls were age and gender-matched with cases), in the period from March 2017 to October 2017. The study was conducted according to national and international ethical guidelines (good clinical practice, Declaration of Helsinki). The protocol was approved by the National Liver Institute Hospital Local Ethics Committee, Menoufia University (NLI-001.09.2017/1), and written informed consent was taken from all subjects. The genetic data obtained from the samples was used completely for the objective of this research. All enrolled individuals were subjected to clinical examination, medical history, laboratory workup including blood picture, some liver and kidney function tests, serum alpha-fetoprotein, and abdominal ultrasound. Only the HCC patient group was subjected to further spiral triphasic CT and/or MRI. All cases were HBs Ag negative. None of the patients had received antiviral therapy. Patients who had infections or malignancy rather than HCC were excluded from this study.

### HPSE SNP genotyping

Genomic DNA was purified from peripheral venous whole blood using ABIOpure™ total DNA extraction kit (Bothell, WA 98021 USA) according to the kit manufacturer’s guidelines. The extracted DNA was kept at − 20 °C until use. *HPSE* rs12503843 SNP was analyzed using polymerase chain reaction-restriction fragment length polymorphism (PCR-RFLP). In brief, genomic DNA was subjected to amplification using PCR running under the thermal conditions: 95 °C for 5 min followed by 35 cycles of 95 °C for 30 s, 55 °C for 30 s, 72 °C for 1 min, and extension final step at 72 °C for 10 min. Sequences of the primers used were as follows: 5′- AAA GCA AAA GGA TGT GAA CAC AAA -3′ (forward), 5′- CTT ACT CTA ACC AAT AAA AAT TAA TGC TAT AGA -3′ (reverse). Subsequently, 1 μg of the amplified PCR product was digested with 5 units of *MnlI* fast digest restriction enzyme (Thermo Fisher Scientific Inc., USA) for 2 h at 37 °C. Then, RFLP products were separated on a 3% agarose gel electrophoresis and visualized by Gel-Doc Imaging System (E-Box VILBER, France). After digestion, two fragments of 261 and 2237 bp were detected in CT genotype, but only 237 bp fragment was detected in CC genotype, and finally, the 261 bp fragment only was detected in TT genotype. Another *HPSE* rs12331678 SNP was genotyped by using a modified ARMS PCR assay (Amplification Refractory Mutation System). Briefly, (A allele) forward (5′-GTA TTT CCT ACA TTA TAG AGT TTG CTA **A**-3′), (C allele) forward (5′-GTA TTT CCT ACA TTA TAG AGT TTG CTA **C**-3′) and common reverse primer, 5′-CAT GAT GAA ACC CCT TCT GTA C-3′ were used for amplification of *HPSE* rs12331678 by ARMS-PCR. In every sample represented by two PCR reactions using A or C allele forward primer, the *HPSE* rs12331678 AA genotype generated a single band with A allele forward primer only, and the CC genotype generated a single band with C allele forward primer only, whereas the two bands were generated in heterozygous CA genotype.

### Data analysis

IBM SPSS computer software package version 20.0. (Armonk, NY: IBM Corp) was used to analyze the obtained data. Qualitative data analysis was calculated using percent and number. The distribution normality was verified by the Kolmogorov-Smirnov test. Quantitative data analysis was done by using mean, standard deviation, and range (minimum and maximum). The results significance was judged at the level of 0.5%. The *P* values, odds ratios (ORs), and 95% confidence intervals (95% CIs) were then determined. Further, the used tests were Fisher’s exact or Monte Carlo correction chi-square test, Mann-Whitney test, Student’s *t* test, and odds ratio (OR). *P* value < 0.5 is considered statistically significant.

## Results

### Characteristics of the study population

The selected attributes of the case-control subjects are mentioned in Table 1. No significant differences were found between patients with HCC and control in terms of sex and age. The values of AFP, TBIL, AST, and ALT increased significantly in HCC patients than in healthy individuals (*P* < 0.001). The hematological parameters show that the values of the hemoglobin (Hb), RBCs, WBCs, platelets, and prothrombin concentration were significantly decreased in HCC than in control groups (*P* < 0.001), while the creatinine and total bilirubin levels were not different significantly among the two groups (*P* > 0.05).

**Table 1:** Selected clinical and demographic characteristics of patients and controls

Variables	Patients (***n*** = 70) Mean ± SD	Control (***n*** = 30) Mean ± SD	Test of sig.	***P***
**Demographic data**				
Age, years	60.40 ± 10.47	60.64 ± 7.81	*χ*^2^ = 0.233	0.898
Sex, *n* (%)	M, 65(92.9%); F, 5(7.1%)	M, 27 (90%); F, 3 (10%)	*t* = 0.128	0.694
HCV carriers, *n* (%)	43 (61%)	0 (0%)		
**Hematological profile**				
HB	12.53 ± 1.61	13.36 ± 13.36	*t* = 2.531	< 0.001*
RBCs.	4.35 ± 0.63	4.65 ± 0.39	*t* = 2.821^*^	< 0.001*
TLC	5.23 ± 1.88	6.61 ± 1.44	*t* = 3.605	< 0.001*
PLT	122.53 ± 60.47	270.27 ± 71.36	*U* = 134.500	< 0.001*
Pro. Conc.	71.97 ± 14.92	95.47 ± 5.04	*t* = 11.711*	< 0.001*
INR	1.28 ± 0.20	1.02 ± 0.04	*t* = 11.711*	< 0.001*
**Biochemical parameters**				
Creatinine	0.91 ± 0.23	1.0 ± 0.15	*U* = 723.50*	0.13
AST	59.14 ± 33.92	32.30 ± 5.93	*U* = 343.0*	< 0.001*
ALT	48.60 ± 33.69	25.77 ± 6.73	*U* = 407.50*	< 0.001*
Total bilirubin	1.34 ± 0.68	1.08 ± 0.13	*U* = 944.50*	0.426
Direct bilirubin	0.57 ± 0.43	0.18 ± 0.08	*U* = 332.0*	< 0.001*
Albumin	3.31 ± 0.62	4.31 ± 0.61	*t* = 7.375*	< 0.001*
AFP	1172.46 ± 2627.92	6.04 ± 1.05	*U* = 199.50*	< 0.001*
**Child-Pugh score**				
A	60.0%			
B	37.1%			
C	2.9%			
**Focal lesion**				
**Size (cm)**	5.08 ± 3.14			
**Number**				
**1**	65.7%			
**2**	20.0%			
**3**	5.7%			
**> 3**	8.6%			

*t* Student’s *t* test, *U* Mann-Whitney test

*Statistically significant at *P* ≤ 0.05

### Genotypic and allelic frequencies of *HPSE* SNPs in the study groups

**Fig. 1:**
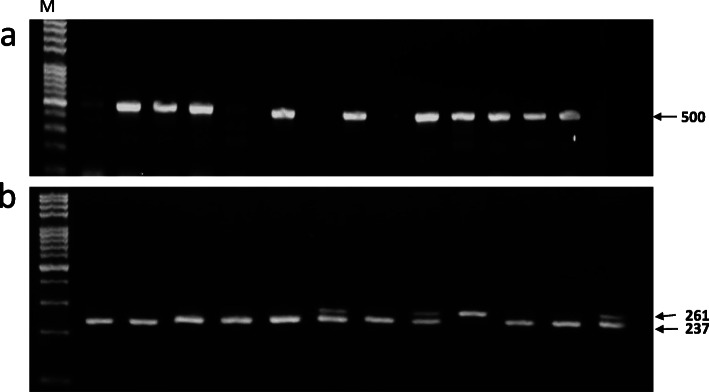
The *HPSE* SNP genotyping from Egyptian patients with HCC and control individuals by agarose gel electrophoresis. **a** genotyping of *HPSE* rs12331678 by ARMS-PCR as the first 2 lanes are CC genotype and the lanes 3 and 4 are CA genotype. **b** genotyping of HPSE rs12503843 by RFLP-PCR; CC genotype product 237 bp, while CT genotype 237 and 261 bp and TT genotype 261 bp

The main goal was to gain the distribution of the *HPSE* polymorphisms within the Egyptian HCC patients. To obtain the knowledge concerning the *HPSE* SNP distribution among the Egyptian HCC patients, initially, the genomic extracted DNA from 30 control subjects were analyzed. The frequencies of rs12331678 genotypes within this control group were 56.7% CC, 43.3% CA, and 0.00% AA, while for the rs12503843, they were 73.3% CC, 26.7% CT, and 0.0% TT (Table 2 and Fig. 1). SNP rs12331678 and rs12503843 in the control group showed to be in Hardy-Weinberg equilibrium (*χ*^2^ tests, *P* ≥ 0.05), which gives the allowance to proceed with the HCC patient genotype distribution. Next, the distribution of *HPSE* polymorphisms within an Egyptian cohort of 70 HCC patients was done. SNP genotyping in the HCC subjects showed that the distributions for the rs12331678 genotypes were 67.1% CC, 31.4% CA, and 1.4% AA, while distributions for the rs12503843 CC, GT, and TT genotypes were 52.9%, 41.4%, and 5.7%, respectively (Fig. 1 and Table 2). The genotype frequencies corresponding to rs12503843 (CC, TT, CT) among the control and the HCC cohort (Fig. 1b) demonstrated to be non-statistical difference (*P* > 0.05), but regarding allelic distribution among the two groups, analysis showed to be a statistically significant difference with a higher prevalence of the unfavorable (T) allele within the HCC group (Table 2). The same analysis was performed for *HPSE* rs12331678; no statistically significant differences were observed between both cohorts either in genotypic or allelic distribution (Fig. 1a and Table 2). Furthermore, the SNPs stratification based on HCV carrier in HCC groups were analyzed, and the results revealed that there were no statistically significant differences between the HCV carrier and non-carrier individuals in the HCC group regarding the *HPSE* rs12331678 and rs12503843 (Table 3).

**Table 2:** Genotypic and allelic frequencies of *HPSE* SNP in HCC and control group

SNP	Patients (***n*** = 70)	Control (***n*** = 30)	OR	95% CI	***χ***^**2**^	***P***
	%	%				
**rs12331678**						
AA	1.4	0.0	-	-	1.674	0.553
CA	31.4	43.3	0.599	0.25–1.44		
CC	67.1	56.7	1.563	0.65–3.76		
**Allele**						
A	17.1	21.7	0.748	0.35–1.59	0.570	0.450
C	82.9	78.3	1.337	0.63–2.84		
**rs12503843**						
TT	5.7	0.0	–	–		
CT	41.4	26.7	1.945	0.76–4.97	3.858	0.128
CC	52.9	73.3	0.408	0.16–1.04		
**Allele**						
T	26.4	13.3	2.335	1.01–5.37		
C	73.6	86.7	0.428	0.19–0.98	4.130*	0.042*

χ^2^ chi-square test

*Statistically significant at P ≤ 0.05

**Table 3:** Stratifications of rs12331678 and rs12503843 in correlation to HCV carrier status in HCC group (*N* = 70)

SNP	HCV positive ***N*** (%)	HCV negative ***N*** (%)	***χ***^**2**^	***P***
**rs12331678**				
CC (*n* = 47)	43 (91.5)	4 (8.5)	5.189	0.129
CA (*n* = 22)	20 (90.9)	2 (9.1)		
AA (*n* = 1)	0 (0.0)	1 (100.0)		
**rs12503843**				
CC (*n* = 37)	33 (89.2)	(10.8) 4	0.230	1.000
CT (*n* = 29)	26 (89.7)	(10.4)3		
TT (*n* = 4)	4 (100.0)	0 (0.0)		

*χ*^2^ chi-square test

To elucidate the role of *HPSE* rs12331678 and rs12503843 gene polymorphisms in the HCC patients’ clinicopathologic status, the association of clinical features and distribution of *HPSE* SNP polymorphisms in HCC subjects were evaluated, including Child-Pugh score, focal lesion size and number, HCV infection (anti-HCV), and the common HCC clinical-pathological features including AFP, ALT, and AST. No significant relation was observed between the *HPSE* rs12331678 and rs12503843 gene polymorphisms and all clinicopathologic status and markers (Tables 4 and 5).

**Table 4:** Association between rs12331678 and clinicopathological features

Variables	rs12331678						***P***
	AA (***n*** = 1)		CA (***n*** = 22)		CC (***n*** = 47)		
	No.	%	No.	%	No.	%	
**ALT**							
Mean ± SD	65.0		48.05 ± 23.08		48.51 ± 38.12		0.652
**AST**							
Mean ± SD	25.0		53.91 ± 24.62		62.32 ± 37.42		0.293
**AFP**							
Mean ± SD	949.0		1357.46 ± 2958.08		1090.62 ± 2517.93		0.695
**Child-Pugh score**							
A	0	0.0	12	54.5	30	63.8	0.345
B	1	100.0	10	45.5	15	31.9	
C	0	0.0	0	0.0	2	4.3	
**Number of hepatic focal lesion**							
1	1	100.0	13	59.1	32	68.1	^MC^*P* = 0.526
2	0	0.0	4	18.2	10	21.3	
3	0	0.0	3	13.6	1	2.1	
> 3	0	0.0	2	9.1	4	8.5	
**Size (cm)**							
Mean ± SD	5.0		5.43 ± 3.47		4.91 ± 3.03		0.796
**HCV**							
Negative	1	100.0	2	9.1	4	8.5	^MC^*P* = 0.129
Positive	0	0.0	20	90.9	43	91.5	

*MC* Monte Carlo

**Table 5:** Association between rs12503843 and clinicopathological markers

Variables	rs12503843						***P***
	TT (***n*** = 4)		CT (***n*** = 29)		CC (***n*** = 37)		
	No.	No.	No.	No.	No.	No.	
**ALT**							
Mean ± SD	45.0–8.29		55.86 ± 43.14		43.30 ± 25.47		0.267
**AST**							
Mean ± SD	70.50 ± 24.72		67.21 ± 67.21		51.59 ± 21.52		0.62
**AFP**							
Mean ± SD	191.20 ± 336.35		1015.56 ± 2441.89		1401.52 ± 2899.74		0.599
**Child-Pugh score**							
A	1	25.0	20	69.0	21	56.8	0.313
B	3	75.0	9	31.0	14	37.8	
C	0	0.0	0	0.0	2	5.4	
**Number of hepatic focal lesion**							
1	3	75.0	19	65.5	24	64.9	^MC^*P* = 0.827
2	1	25.0	4	13.8	9	24.3	
3	0	0.0	2	6.9	2	5.4	
> 3	0	0.0	4	13.8	2	5.4	
**Size (cm)**							
Mean ± SD	6.38 ± 4.46		4.71 ± 3.22		5.22 ± 2.97		0.325
**HCV**							
Negative	0	0.0	3	10.4	4	10.8	^MC^*P* = 1.0
Positive	4	100.0	26	89.7	33	89.2	

*MC* Monte Carlo

## Discussion

The most common human genome sequence variation is the single nucleotide base substitution, commonly named as an SNP. SNP is a stable nucleotide variation in sequence with an occurrence of more than 1% in at least one population [29]. A single variant effect is probably insignificant, but combinations of different SNPs, either in the same gene or among distant genes, could corporately contribute to disorder occurrence. Studies on genetic associations have begun for studying the SNP effect on the disease outcome, as it changes the phenotypic expression of a recognized gene making the individual more susceptible to a specific disease [30].

Hepatocellular carcinoma (HCC) is among the most frequently occurring malignancies and a prominent cancer cause associated mortalities worldwide. The HCC etiology remains mostly elusive. Presently, the well-known HCC risk factors are chronic viral hepatitis, liver cirrhosis, aflatoxin exposure, alcohol consumption, and smoking [31, 32]. However, a few fractions of individuals with recognized risk factors finally develop HCC, implying that other environmental and genetic mediators may be participating in the development of HCC. In this manner and considering that there was not enough information on genotypes distribution of *HPSE* SNPs for Egyptian HCC patients, the evaluation of the frequency of *HPSE* rs12331678 and rs12503843 in a number of Egyptian HCC patients and control individuals was at high interest.

In the present study, 70 HCC patients and 30 control subjects were genotyped for two SNPs of *HPSE* gene (rs12331678 and rs12503843). Analysis of allele genotype frequencies of rs12331678 revealed that no significant difference was revealed among the HCC patients and control group regarding the frequencies of different genotypes. On the other hand, the unfavorable (T) allele of rs12503843 was found at a high frequency in the HCC group, given a higher prevalence of favorable rs12503843 (C) allele in healthy individuals when compared to HCC patients. This result suggests that rs12503843 may be significantly correlated with HCC susceptibility in Egyptian individuals.

The possible mechanisms which explained the association between the HCC risk and *HPSE* rs12503843 may include the functional role of this SNP to serve as a marker in tight linkage disequilibrium (LD) with other functional SNPs in the 3′UTR region of *HPSE* [33]. Ostrovsky et al. have stated that the rs4693602 SNP, which is present in the 3′UTR distal part of the *HPSE* gene, was correlated with multiple myeloma (MM) disease and may alter the expression of the HPSE gene. The intronic rs12503843polymorphism in tight LD together with rs4693602 might act as genetic markers, possibly because they are located in downstream of the *HPSE* 3′UTR region [23].

The associations between the HCC risk and these SNPs were assessed with HCV carrier status stratification. There was a non-significant interaction between rs12331678 and rs12503843 and HCV carrier status, indicating that this status did not modify the HCC susceptibility. Finally, the association between the SNPs and clinicopathological features was examined; however, we could not show statistically significant relevance, and this may be attributed to the small population size of our pilot study.

On the contrary to the findings by Ostrovsky et al. [23], no relationship was detected between the SNP rs12331678 and the occurrence of HCC (*P* = 0.553), but our finding comes in accordance with the results by Winter et al. [28]. A possible explanation is that different genetic mechanisms of the susceptibility of different diseases might be involved in population-specific variations. However, our negative findings could be attributed to genetic variation influence among ethnic groups, e.g., differences in the pattern of LD or allele frequencies of *HPSE* between populations. Our results come in agreement with recently published data by Yu et al. who stated that the *HPSE* rs12503843 (T) allele was more susceptible to HCC development in the Chinese population [33].

## Conclusion

The current pilot study provides, for the first time, *HPSE* gene SNP characterization among Egyptian patients diagnosed with HCC and suggests the associations between the *HPSE* rs12503843 only with the HCC development. Therefore, results of our pilot study offer the rationale for further larger trials to elucidate clinical significance and importance of the HPSE gene polymorphisms in HCC pathogenesis. Furthermore, a direct connection between heparanase expression and HCC-associated variants or function should be investigated more intensely.

## Data Availability

Seventy HCC cases and thirty control subjects were recruited from the National Liver Institute, Menoufia University, Egypt.

## Abbreviations

HCC: Hepatocellular carcinoma
HPSE: Heparanase
SNP: Single nucleotide polymorphism
MAP: Multiple antigenic peptides
HCV: Hepatitis C virus
HBV: Hepatitis B virus
LD: Linkage disequilibrium
OD: Odds ratio
MM: Multiple myeloma
BM: Basement membrane
ECM: Extracellular matrix
PCR: Polymerase chain reaction
RT-PCR: Real-time PCR
TMAs: Tissue microarrays
CI: Confidence interval
